# Exploring experiences of people participation activities in a British national health service trust: a service user-led research project

**DOI:** 10.1186/s40900-019-0140-8

**Published:** 2019-01-28

**Authors:** Adrian Curwen, Jane Fernandes, Rachael Howison, Paul Binfield, Frank Rohricht, Domenico Giacco

**Affiliations:** 1 East London NHS Foundation Trust, People Participation Team, London, E1 8DE UK; 2 East London NHS Foundation Trust, London, E1 8DE UK; 3 Unit for Social and Community Psychiatry (WHO Collaborating Centre for Mental Health Service Development), Queen Mary University of London, London, E1 4NS UK; 4 Unit for Social and Community Psychiatry, Newham Centre for Mental Health, Glen Road, London, E13 8SP UK

**Keywords:** Mental health, Service user involvement, People participation

## Abstract

**Background:**

People participation teams are being established in many British NHS Trusts. They support active service user involvement in staff recruitment interviews, in evaluations of care and in programmes to improve services. No studies exploring experience of people involved in these activities are available in published mental health literature. In this project we explored benefits experienced and suggestions for improvement provided by service users.

**Methods:**

The design, materials and methods of the project were developed in workshops including *N* = 15 service users. Three service user researchers were trained to carry out research interviews and qualitative analysis of the data. They were responsible for the management of the project on a day-to-day basis with weekly supervision by academic researchers and authored this paper. The service user researchers interviewed people with at least 1 year of experience of being involved in a People Participation team activities. Interviews were based on a topic guide, which was developed in workshops with a larger number of service users (*N* = 15) and explored reasons for joining the group, how participation helped recovery and suggestions for service improvement.

**Results:**

Fifteen service users were recruited and interviewed. Reasons for joining the group were identified: to “give back” to the service, to influence service change, curiosity, desire to meet like-minded people and to structure the day. Benefits reported included: sharing experiences, improving self-confidence, feeling valued, having a better understanding of services, overcoming personal fears, and developing better coping mechanisms for psychological difficulties. Being involved in People Participation activities helped to gain or refresh listening and interpersonal skills, communication skills, public speaking and creative skills and to develop better ways to cope with conflict. Suggestions for improvement were focused on changing staff attitudes, further promoting participation (e.g. through websites), simplifying payment procedures and establishing a moving-on support system to help people to access regular employment and gain full social inclusion.

**Conclusions:**

Our findings showed that People Participation initiatives can have benefits at least for some patients and help their recovery through a positive effect on self-confidence, providing room for feeling valued and for obtaining or refreshing personal skills. This provides support for the development and refinement of People Participation Teams and for larger scale research to test their effects.

## Lay summary

Many NHS Trusts in England established People Participation teams. These teams support service user participation in Trusts’ activities. Activities include staff recruitment interviews, evaluations of care and programmes to improve services. Many service users report benefits from working with People Participation teams, but there is little research on these teams so far.

Service users led in developing the design and the interview schedules for this research and three of them carried out the practical research activities, the analysis of data and authored this paper, under the supervision of experienced researchers.

People who had at least 1 year of experience of People Participation Teams were interviewed for this research. The interviews explored reasons for joining these teams, overall experience of involvement and suggestions for improvement.

Fifteen service users were interviewed. They said they had joined the People Participation teams for different reasons: to “give something back” to the Trust which had provided care for them; to influence positive change in the care of other people; c) to satisfy their curiosity; to meet like-minded people; and to give some structure to their day.

They found it beneficial to share experiences, and said that their involvement in PP improved their self-confidence, made them feel valued, understand services better, overcome personal fears, and develop better ways to cope with their mental health problems.

They felt that People Participation activities helped them to develop their interpersonal, communication, public speaking and creative skills and learn how to better cope with conflicts.

Suggestions were made to further promote People Participation teams, simplify payment procedures for involvement and help people to move on from participation in the activities of these teams to regular employment and full social inclusion.

The benefits that were found in this small study can be seen as initial support for further developing the model of People Participation teams. The benefits and shortcomings of these teams will, however, require further larger scale studies.

## Background

Across the United Kingdom People Participation teams are being established in NHS Trusts in order to enable active participation of service users, carers and the public in NHS Trust activities. The activities in which People Participation Teams facilitate the participation of service users include service planning, training to clinicians, audits, research and staff recruitment panels [1].

The involvement in these People Participation Groups of mental health service users has been anecdotally reported by many participants as beneficial. However, there is little systematic evaluation of what are the benefits experienced by participants and what are their preferences on how these teams and their service should be organised.

Building on previous experiences of collaboration and co-production of research projects with service users and carers [2, 3] we designed an evaluation of a people participation group in a mental health Trust.

Our project adopted a novel approach, going beyond the usual paradigms and methodologies of patient and public involvement in research. Normally service users are involved in research in an advisory capacity or as additional members of an established research group [4–10].

In our project a group of service user researchers were recruited and trained to carry out the research interviews and the day-to-day research activities with weekly supervision by senior academics.

The project was focused on experiences of service users joining People Participation teams and on the following questions:

- Why do service users join People Participation activities?
- How can People Participation activities help their recovery?
- What are service users’ suggestions for improvements of People Participation activities?

## Methods

### Project preparation

This project was funded by a grant of the Centre for Public Engagement of Queen Mary University of London. It was named PRIDE (Participation, Engagement, Involvement, Recovery and Experience). The funding application was developed by Giacco, Rohricht and Binfield, who is head of People Participation at East London NHS Foundation Trust (ELFT) and has previous lived experience of mental health problems and of using mental health services.

We made the choice of having a research team almost entirely including service users (although with supervision from more experienced professionals) as this was in line with the ethos of the people participation scheme. We aimed to create an absence of any perceived power dynamic and hierarchy between the interviewers and participants and allow participants to feel more comfortable in expressing their views.

The project preparation included recruitment of service user researchers, co-production of study materials and application for study approval.

#### Recruitment of service user researchers

A job advert was produced in order to recruit three service user researchers for this project. A seven-month part-time job was offered. The allowance was in line with that permitted by the United Kingdom Department for Work and Pensions in order to not impact on benefits and, hence discriminate people who were receiving benefits from potential participation.

The interested service users (or carers) had to meet the following criteria to be invited to an interview:

1. Experience of using mental health services within the last year or supporting someone (family member or friend) who has used mental health services within the last year;
2. Good understanding of written and spoken English;
3. Being able to use a computer for basic word processing and accessing emails;
4. Being able to travel to East London NHS Foundation Trust headquarters where the project was based.

Desirable criteria were:

1. An understanding of the research process (but no previous research experience);
2. Knowledge of how mental health services are organised;
3. Knowledge of how People Participation opportunities work.
4. Experience of being involved in People Participation opportunities in a NHS Trust.

Curwen, Fernandes and Howison were employed and worked on the project as service user researchers. These three service user researchers were one male and two females and all have been involved in People Participation activities.

#### Co-production of research materials

The first step was to adapt a training module to quantitative and qualitative interviewing. Our purpose was to make it user-friendly and understandable to people and service users who have an interest in research but no previous research experience or formal qualifications.

This was achieved through gradual modification of a training module originally used for formally trained researchers. This training module was read by the three service user researchers, discussed during team meetings and modified in language and graphic presentation by the three employed service user researchers in order to remove jargon and over-technical terms. The final training module is available on request contacting the authors.

The three service user researchers and the academic lead (Giacco) then organised and chaired two workshops with a higher number of service users who had previous experience of involvement in a People Participation Team (*N* = 15).

During these workshops the research aims of the project were specified and a semi-structured topic guide was developed, which was then used for the qualitative interviews.

The topic guide included: a) Reasons for joining People Participation Group; b) How People Participation Groups can help in personal recovery; c) Skills refreshed or obtained through participation; d) Experience of support provided; e) Suggestions for improvement.

#### Application for study approval

The application and study materials were produced by the service user researchers under the supervision of the academic lead and were approved by the East London NHS Foundation Trust ethics committee for service evaluation projects.

### Service evaluation activities

We carried out qualitative interviews with service users who were had at least 1 year of experience of involvement with the People Participation Team (qualitative interviews).

#### Data collection

Three service user researchers (Curwen, Fernandes and Howison) have carried out the day-to-day research activities. Participants were recruited through e-mail invitations to those service users who had contributed to the People Participation team activities for more than 1 year. Interested participants self-referred to the service evaluation team and contacted the service user researchers.

Activities carried out by service user researchers included contacting potential participants, explaining the project, obtaining written informed consent and carrying out recording and transcribing of the interviews. The first interview of each service user researcher was carried out in the presence of the academic lead (Giacco) who would give feedback to the service user researchers after its completion. This may have somewhat influenced the setting of the first interview, the behaviour of the interviewers and participants and consequently the material. However, it was strongly felt within the preparatory workshops that attendance of an experienced researcher during the first interview would be important to increase the confidence of service user researchers (who had never interviewed people for research) and to provide an opportunity for direct feedback.

Regular individual and group supervision took place thereafter. Service user researchers could rely on availability of Giacco and Röhricht to discuss any clinical issue or concerns arising during the interviews.

#### Data analysis

The analysis was carried out in a group with the participation of the academic lead (Giacco), a senior clinician and academic (Röhricht) and the People Participation Team lead (Binfield).

The analysis followed the principles of inductive content analysis. This allowed the identification of trends and patterns in the data by identifying recurrent meanings and themes in the responses [11, 12]. The analysis was driven by the service evaluation questions. The study team assessed the data, and added notes and headings in the text in order to describe the content. The process of grouping similar codes under themes followed. Saturation of themes was decided upon by the study team in group discussion. The identified themes and subthemes were then checked and refined in group discussion, using a simplified version (i.e. not including independent analysis by each researcher) of a standard content analysis approach [13]. The three service user researchers carried out the analysis and discussed codes and themes with Giacco. Their previous positive experience of taking part in People Participation activities was discussed as a source of potential bias before the analysis. Giacco is a clinical and academic psychiatrist at ELFT, who however, has no formal links with the People Participation Team (PP).

## Results

### Fifteen participants were interviewed

Seven of them were female (46.7%). Ethnic group distribution was 33% White Caucasian; 27% Black African/Caribbean, 20% mixed ethnic group, 13% Asian. One participant preferred not to define her/is ethnic group. Eight participants were older than 44 years old (53.3%) and two reported to have a learning disability (13%).

**Fig. 1:**
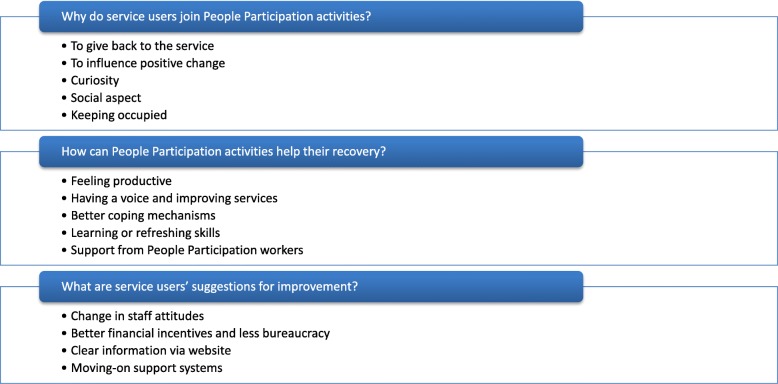
Summary of identified themes

The emerging themes are reported in the following paragraphs in response to the service evaluation questions and summarised in Fig. 1.

#### Why do service users join people participation activities?

##### To “give back” to the service

A number of service users regarded the participation in the activities of a NHS Trust as a way to devote their time and efforts to an organisation that had helped them in the past.

*“I felt a sort of passion in wanting to help improve things. I felt sort of like a need to pay back some of the really great sort of professionals I met across the years who’d helped me out”.* **P11**

##### To influence positive change

Another factor that motivated the choice to join People Participation activities was the willingness to improve the care for their fellow service users. Participants felt that participating in Trust activities put them in the position of being able to improve care.

*“I think the move towards greater patient engagement, if you like, with their own treatment and the way that they are dealt with by the NHS, I think participation is a huge step towards that…”.* **P14**

*“I’ve had quite a few staff remark to me that I’ve changed their attitude of service users and service user involvement in peer support and that sort of thing. So I think I’ve changed some attitude there”.* **P11**

##### Curiosity

Some service users reported that they were motivated by curiosity and a willingness to find and face new challenges.

*“So really it was the PPL lead… and s/he came along and, I’m not even sure how it happened, but I got involved and I started enjoying it. It was hard at first, especially talking to many people. Very scary and very shaky, but s/he kind of made it a lot better. S/he’s really good at her/is job. S/he really looked after me, you know, and I feel there’s been progress since I first started.”* **P7**

##### Social aspect

Another aspect that attracted service users to take on PP was the possibility to meet like-minded people in a context, which is different from standard therapeutic groups.

*“I come to the meetings and I look forward to coming… because it’s a change from that routine of hanging around with people (and) doing things that are not going to help them in their mental state.”* **P1**

*“I needed to be involved in getting to know some other people.”* **P2**

##### Having structure to their day and being occupied

Some participants reported that being involved in People Participation was a way to keep themselves occupied and having a structure to their day. This may be particularly important when they are not engaged in work or family activities. Understanding other people’s problems might help to cope with one's own psychological difficulties.

*“People Participation has turned my life around in the last 2 years I have been doing it. It gives me something to do. It involves me in aspects of other people’s illnesses, understanding other people’s illnesses.”* **P5**

*“I feel that I am happy…mental state as well because I feel happier when doing participation.”* **P4**

#### How can people participation groups help recovery?

##### Being productive

Participants mentioned that doing something actively to make a difference to other people’s care can be a positive experience and improve perceptions of themselves. An important part of this is feeling respected, valued and appreciated by people with similar experience of a mental health condition.

*“It helped with my recovery greatly. Sort of helping other people and feeling productive and putting a positive end to a negative set of experiences. It’s all, sort of, been great.”* **P11**

*“To be able to express my views, meet like-minded people who have gone through the same thing.”* **P4**

*“You get to connect with people and it’s so lovely when people come up to you and say “I love coming here because you are here as well” and, you know, that sort of thing. Just to be you.”* **P8**

##### Having a voice and improving services

Feeling respected and valued for their judgement and particularly having their opinions listened to when making major decisions for the services (e.g. recruitment of staff) or influencing staff attitudes can have a positive effect on self-esteem.

*“It made me more empowered because I was sitting on panels and I was having a say of who comes in and who doesn’t come in”* **P1**

*“Getting involved… taking part, having a say, being listed to, being educated…”* **P1**

*“I’ve had quite a few staff remark to me that I’ve changed their attitude of service users and service user involvement in peer support and that sort of thing. So I think I’ve changed some attitudes there.”* **P11**

##### Improving coping mechanisms

Having more direct contact with services from the “inside” can improve the way in which some participants respond to stress, facilitate learning and adoption of positive coping mechanisms.

*“I ain’t had drugs, drunk alcohol for 17 years, I haven’t smoked cigarettes for 12 years… it’s made me more self-aware of how you can end up back in hospital again or in trouble with the law if you don’t do things that are positive rather than negative.”* **P1**

*“It’s helped me because it’s made me think about what are the good things in life and what are the bad things in life and what’s going to keep me well and safe and keep me from going back to hospital again.”* **P1**

##### Learning or refreshing skills

Many participants reported that taking part in people participation activities was a way to acquire or refresh skills and do things that they believed were not (or not anymore) possible for them. Participants talked about listening skills, general communication skills and more specific abilities such as public speaking skills.

*“I’ve learnt so much from going to the meetings, you know, talking and listening to other people, so I’ve learnt a lot, and I’ve got sort of self-respect and my say back, which I didn’t have before”* **P5**

*“It trains you to develop your skills set. That was very attractive to me.”* **P7**

*“I think being able to express yourself, especially when I do talks with new nurses or new social therapists, they really want to hear the service user’s view and see the other side. Not just the things they are trained in. Not just the things that are passed down, but the service user’s view is the reality. The fact that I was a patient made my views more important.”* **P7**

##### Support from people participation workers

Participants were keen to emphasise the help received from professionals (often with a personal experience of a mental health condition) who work in people participation teams. The support of these professionals can help service users to manage their fears and to push personal boundaries, to stay up to date with local events and to overcome individual problems when engaging with activities. The role of these professionals as companions rather than clinicians and the feeling that they are available and trusted is important for a positive experience of service users.

*“Yes, s/he has been really good. I’ve needed to lean on her/im quite a bit. Especially when writing any script or doing any talk, the fact that s/he’s there makes it much easier. I can get all the information that I need and s/he really supports me. S/he does a wonderful job. S/he has great qualities, you know. So I wouldn’t be able to do the stuff I’ve done without her/him.”* **P7**

*“Our People Participation Lead is probably the best one and I wouldn’t want anyone else. I can talk to her/im about anything. S/he is down to earth, human. S/he’s a lovely lady and I can go to her/im whenever I like.”* **P8**

*“I set myself boundaries because I guess we all live in our own safety nets when you have mental illness. S/he actually makes me go to the edge and sometimes over. And when I do that, I feel, you know, like, ‘wow, I’m so glad I did that. Can I do that? I can really do that’ you know.”* **P8**

*“People Participation Leads should be on ward rounds. You can talk to People Participation Leads about things you wouldn’t talk to a doctor about.”* **P10**

#### What are service users’ suggestions for improvement of people participation activities?

All the study participants’ suggestions focused directly or indirectly on strategies to promote the access or retention of more service user in People Participation Activities.

##### Change in staff attitudes

Some participants reported that they felt that their participation in service activities was not valued enough. They mentioned that some clinicians or service managers would not adequately listen to their opinions and consider them when making decisions about the service. This can occur for example in interview panels for staff recruitment. They asked for awareness training to change attitudes of some staff members.

*“My first interview panels staff were condescending… but after doing more with them they got used to me and they changed their attitude.”* **P14**

*“Some of the staff … still don’t see the lived experience as having any sort of value and they think that mental health patients are completely unqualified to comment.”* **P11**

##### Better financial incentives and less bureaucracy

Some participants felt that the financial incentives to be involved were too scarce and increasing the retribution for service user involvement may be a way to get more people interested. For others the problems were more related to the difficulties in receiving payment due to complex bureaucratic processes and the many forms they have to complete to receive recognition for their contribution.

*“I guess all these form-filling and stuff to get paid makes it complicated”* **P8**

*“Payment system is quite complicated with forms going backwards and forwards… getting signed off here and there by different people.”* **P11**

##### Clear information via website

Disseminating clear and user-friendly information might help identifying other service users interested in PP and the engagement of people who are already involved with the People Participation Team. A visible and accessible website with clear content might serve this purpose.

*‘This is the 21st century, a web page, details of events… can be updated as soon as an event is cancelled, or advertise opportunities.’* **P2**

##### Moving-on support systems

Participants suggested that “moving-on support systems” should be part of People Participation Team programmes. This can help people who have engaged and benefitted from PP activities to progress to the next step and pursue their goals in terms of competitive employment and social inclusion.

*“Moving on, I think there should be a careers advice aspect to it. I don’t think it should be mandatory because obviously the thing with working within mental health, everyone is moving at their own pace.”* **P11**

*“I think there should be some sort of a monthly job club and moving-on, sort of a support group for people who are working on sort of where is this heading.”* **P11**

## Discussion

This study offered two types of outputs of high interests in the field of service user involvement in health care and research.

One important output is that its findings were among the first ones providing insights on experience of People participation activities in NHS Trusts. This is a still understudied topic and our paper, to our knowledge, is the first contribution to the scientific literature, although the debate on how to carry out PP activities has been going on for a long time in conferences and fora dedicated to service user involvement. A number of benefits such as increasing self-confidence, reducing social isolation and obtaining or refreshing personal skills were mentioned by the participants, which could be a focus for further development of these services. Suggestions for improvement provided some potentially useful strategies to increase availability and knowledge of People Participation activities among a larger number of service users.

These findings are new in the literature and can be the first step to guide quality improvement initiatives for similar services in the NHS.

A second important output is beyond the mere analysis of the service evaluation data. The project produced a training into research interview methods which enabled service users to carry out research activities with limited supervision. This may pave the way to similar initiatives which can improve understanding, interest and involvement of service users and the public in research.

### Strengths and limitations

The strengths of this project lie in his ethos which led to the enablement of service user researchers to carry out and successfully complete service evaluation activities in a limited timeframe (7 months, from January 2017 to July 2017). Active involvement of service user in research is increasing in many areas of healthcare [14]. However, our project is still one of only few research projects in which service users had a leading role in all research activities from designing the study to writing up the findings. These activities produced meaningful and helpful findings that have been used for local service improvement.

The limitations are related to the small size of the sample and to the recruitment carried out in a specific area (East London). Moreover, the service users interviewed in this qualitative research had been in contact with the People Participation team for more than 1 year. We made this methodological choice in order to interview people with a well-rounded experience. However, it is possible that people who had a shorter engagement with People Participation activities or disengaged from it might have had more negative experiences and, hence, more suggestions for improvement. The three service user researchers also had positive experiences of being part of the activities of the People Participation team which may have caused a bias towards positively interpreting experiences of research participants during the analysis.

All these limitations suggest caution when considering the generalisability of these results. Moreover, it needs to be stressed that service user researchers, despite the training and supervision received, cannot be considered as experienced or formally treated research interviewers, which may have affected our findings too.

Finally, due to financial and time constraints, we were unable to carry out a formal evaluation of service user researchers’ experience whilst carrying out research activities, which would have been of value in informing further similar initiatives.

### Implications

Our findings offer an insight, which was so far missing in the literature, on the role and benefits of People Participation activities in NHS Trusts. Despite our results are to be considered initial and need to be confirmed in larger scale studies, These activities can help service users not only to have an active involvement in service improvement, but also to reduce their social isolation and to improve their self-confidence and skills, helping social integration, potentially being a gateway to competitive employment. To achieve full benefits, awareness of these activities should be increased among clinicians and all service users and bureaucratic procedures should be made as uncomplicated and user-friendly as possible.

This service evaluation project adopted a rarely used methodology for service user involvement in research, adopting a service user-led model. This model should itself be evaluated with systematic methods, which was beyond the scope of our research. Yet, it might attract interest in international research as it is in line with the ethos of recovery focused mental health services. Previous studies mentioned benefits of participation in research on people’s recovery, either if they take part as interviewees or with a more active role as advisors or stakeholders. Benefits mentioned were feeling listened to and having a voice in improvement of care [4–11, 14, 15].

An active involvement in research can have additional benefits for people with mental health problems. Training and exercising interview skills can help confidence in interacting with other people and can be transferable skills that can be used in other work endeavours. Being involved in data analysis can help the confidence with computational or conceptual skills which service users may not have had the chance to practise if outside of regular or part-time employment.

Other researchers, service managers or clinicians may be interested in using a similar methodology to evaluate services. For similar projects a regular academic and clinical supervision should be ensured. Academics and clinicians involved in these types of studies need to strike a good balance. It is important to provide autonomy and empowerment, whilst helping service user researchers to manage the stress and objective difficulties of carrying out interviews with a vulnerable population of participants.

Further research or service evaluation activities should also explore experience of People Participation activities in specific groups of service users, for example young or elder people using mental health or other types of health services.

## Conclusions

Our findings gave insight on potential benefits of People Participation teams for their users in terms of increasing self- confidence, feeling of being valued and having a say on their care and obtaining or refreshing skills which are of relevance for their life. These expected benefits provide hypotheses for benefits of People Participation teams which need to be tested in larger and higher quality studies.

Another important learning point of this project is that it showed that mental health service user-led research activities are feasible, and can lead to valuable results for service improvement.

This model for research might be particularly helpful for the evaluation of health care initiatives which focus on the principles of recovery and service user involvement.

## Abbreviation

NHS: National health service
