# Correction to: Exploring experiences of people participation activities in a British national health service trust: a service user-led research project

**DOI:** 10.1186/s40900-019-0146-2

**Published:** 2019-02-28

**Authors:** Adrian Curwen, Jane Fernandes, Rachael Howison, Paul Binfield, Frank Rohricht, Domenico Giacco

**Affiliations:** 1 East London NHS Foundation Trust, People Participation Team, London, E1 8DE UK; 2 East London NHS Foundation Trust, London, E1 8DE UK; 3 Unit for Social and Community Psychiatry (WHO Collaborating Centre for Mental Health Service Development), Queen Mary University of London, London, E1 4NS UK; 4 Unit for Social and Community Psychiatry, Newham Centre for Mental Health, Glen Road, London, E13 8SP UK

**Correction to: Res Involv Engagem (2019) 5: 5**

**https://doi.org/10.1186/s40900-019-0140-8**

In the publication of this article [1] there is an error in the Results section in the sub-section ‘Better financial incentives and less bureaucracy’.

The error: “Some participants felt that the financial incentives to be involved were too scarce and increasing the retribution for service user involvement may be a way to get more people interested.”

Should instead read: “Some participants felt that the financial incentives to be involved were too scarce and increasing the remuneration for service user involvement may be a way to get more people interested.”

This has now been included in this correction.
